# Resistance status of *Anopheles gambiae* s.l. to insecticides following the 2011 mass distribution campaign of long-lasting insecticidal nets (LLINs) in the Plateau Department, south-eastern Benin

**DOI:** 10.1186/s12936-020-3116-0

**Published:** 2020-01-15

**Authors:** Arthur Sovi, Renaud Govoétchan, Razaki Ossé, Come Z. Koukpo, Albert S. Salako, Thomas Syme, Rodrigue Anagonou, Augustin Fongnikin, Udoka C. Nwangwu, Frédéric Oké-Agbo, Filémon Tokponnon, Gil Germain Padonou, Martin Codjo Akogbeto

**Affiliations:** 1 Centre de Recherche Entomologique de Cotonou (CREC), Ministère de la Santé, 06BP2604, Cotonou, Bénin; 2 Faculté d’Agronomie, Université de Parakou (UP), BP123, Parakou, Bénin; 3 Disease Control Department, Faculty of Infectious & Tropical Diseases, The London School of Hygiene and Tropical Medicine, Keppel Street, London, WC1E 7HT UK; 4 Ecole de Gestion et d’Exploitation des Systèmes d’Elevage, Université Nationale d’Agriculture, BP 43, Kétou, Bénin; 5 Faculté des Sciences et Techniques (FAST), Université d’Abomey-Calavi (UAC), BP 32, Abomey-Calavi, Bénin; 6 National Arbovirus and Vectors Research Centre (NAVRC), 33 Park Avenue, GRA, PMB 01573, Enugu, Enugu State Nigeria; 7 Statistics and Actuarial Science Department, College of Liberal Arts and Sciences, The University of Iowa, Iowa City, IA 52240 USA; 8 National Malaria Control Programme, BP323, Cotonou, Bénin

**Keywords:** MIILDs, Efficacy, Resistance, *Anopheles gambiae* sensu lato

## Abstract

**Background:**

In 2011, Benin’s National Malaria Control Programme (NMCP) organized a nationwide mass distribution campaign of LLINs throughout the country. Following this intervention, it was important to assess whether the level of susceptibility of malaria vectors to insecticides had remained the same as compared to the pre-intervention period. The current study investigated this.

**Methods:**

Larval collections were conducted in Ifangni, Sakété, Pobè and Kétou districts located in Plateau department, Southeastern Benin before (2009) and after (2012–2013) LLIN distribution. *Anopheles gambiae* sensu lato (*s.l*.) larvae from the 4 study districts were reared to adulthood and WHO susceptibility tests were conducted. The insecticides tested were deltamethrin (0.05%), permethrin (0.75%), bendiocarb (0.1%) and DDT (4%). Molecular species identification as well as, the characterization of the *kdr* L1014F mutation were also performed in the *An. gambiae s.l.* complex using PCR method.

**Results:**

Overall, a significant decrease in mortality rates of *An. gambiae s.l*. to deltamethrin (0.05%), permethrin (0.75%) and DDT (4%) was observed post-LLIN distribution, respectively: (100% vs 80.9%, p < 0.0001), (77.5% vs 70%, p = 0.01) and, (47.8% vs 4.4%, p < 0.0001). By contrast, susceptibility of vectors to bendiocarb (0.1%) remained the same (100% mortality in the WHO susceptibility tube tests) pre- and post-intervention. An increase in the *kdr* L1014F frequency was observed post-LLIN distribution [F(*kdr*) = 0.91)] compared to the pre-intervention period [F(*kdr*) = 0.56], p < 0.0001. *Anopheles coluzzii* and *An. gambiae* were the two molecular species identified in the study area.

**Conclusion:**

The decrease susceptibility to pyrethroids and DDT as well as, the increase in the frequency of the *kdr* L1014F mutation after the intervention stressed at the time, the need for the development and implementation of effective insecticide resistance management strategies. At present, an update of the vectors resistance status in the area is also necessary for decision-making.

## Background

In sub-Saharan Africa, the major vector of *Plasmodium falciparum*, the parasite responsible for the most severe form of human malaria, is *Anopheles gambiae* sensu lato (*s.l*.) [1]. This highly anthropophilic mosquito comprises 8 sub-species among which *An. gambiae* sensu stricto, *Anopheles coluzzii* and *Anopheles. arabiensis* are the major malaria vectors in Africa [2]. The vector control strategy of Benin’s National Malaria Control Programme (NMCP) relies mainly on the distribution of LLINs and indoor residual spraying (IRS). With the financial support of the President’s Malaria Initiative (PMI) of the US government and the World Bank, the NMCP launched a national mass LLIN distribution campaign in 2011 to ensure universal coverage of the population (1 LLIN for every 1.8 people) [3]. LLINs are an excellent means of providing personal and community protection from malaria [4, 5]. Until very recently, pyrethroids were the only insecticide class used for impregnation of LLINs, owing to their rapid action, excito-repellent effects, effectiveness at low doses and low toxicity to humans [6]. Unfortunately, pyrethroid resistance in malaria vectors has emerged and spread rapidly in several parts of Africa, including Benin [7–11], Burkina Faso [12], Cameroon [13], Côte d’Ivoire [14, 15] and, Kenya [16]. In Benin, *kdr* L1014F mutation as well as metabolic enzymes such as CYP450s, CYP6P3 and CYP6M2 [10, 11, 17, 18] are implicated in malaria vector resistance to pyrethroids.

Distribution of Olyset^®^ nets, a polyethylene 150D LLIN impregnated with permethrin (2%), was carried out by the NMCP in Benin in 2011 with the aims of achieving universal coverage of populations-at risk. It is possible that this expansion of pyrethroid-based vector control may have eliminated susceptible mosquitoes in favour of resistant ones, thus increasing levels of pyrethroid resistance in malaria vector populations [19]. Considering this, the aim of the current study was to monitor changes in insecticide susceptibility and the frequency of the *kdr* L1014F mutation in the natural populations of *An. gambiae s.l.* before (2009) and after (2012–2013) the distribution of LLINs in 4 districts of the Plateau department, South-East Benin.

## Methods

### Study area

**Fig. 1:**
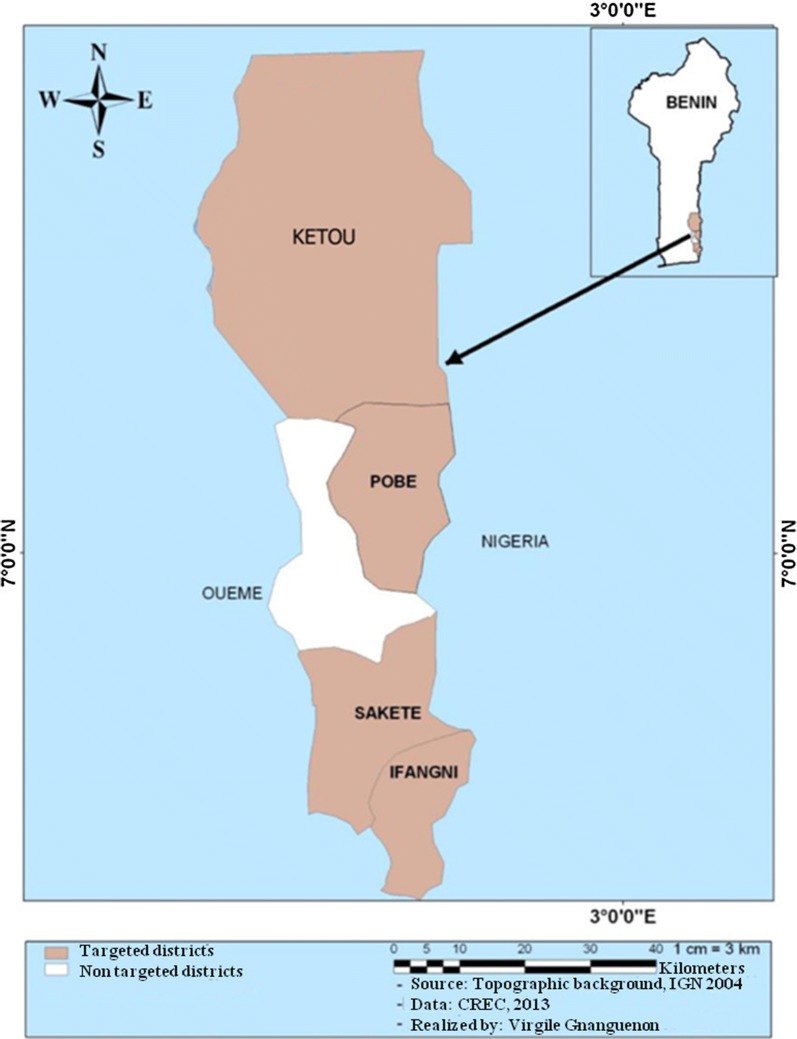
Map showing the study area

The study was performed in Ifangni, Sakété, Pobè and Kétou, 4 districts of the Plateau department, Southeastern Benin (Fig. 1). As of 2013, this department had an area of 3264 km^2^ and a total population of 624,146 inhabitants [20]. The climate is of the Guinean type with two rainy (March–July and, September–November) and, two dry (August and, December–February) seasons occurring annually. There are two cropping periods during the year that coincide with the rainy seasons. Agriculture is the main activity of populations in the Plateau department.

### Collecting mosquito larvae

Field visits were organized between May and July in search of the breeding sites of *An. gambiae s.l.* in 2009 and 2012–2013 respectively before and after the LLIN distribution which occurred in July 2011. In each district, the sampling was performed in 2–3 villages that were the same pre- and post-intervention. Once breeding sites were identified, larvae and pupae were collected from the surface of the water using a larval dipper. The harvested larvae were then reared to adulthood at CREC insectary.

### WHO susceptibility testing

Susceptibility of 3–5 day old female *An.* gambiae s.l. were assessed through the World Health Organization (WHO) tube test method [21]. All tests were performed with papers impregnated at the diagnostic dose recommended by the WHO: deltamethrin (0.05%), permethrin (0.75%), DDT (4%) and bendiocarb (0.1%). For each insecticide, mosquitoes were introduced into four tubes lined with insecticide-impregnated paper in batches of 25 and exposed for 1 h. A fifth batch of mosquitoes was exposed to a tube lined with untreated paper which served as a control. During exposure to the insecticide, the number of mosquitoes’ knocked down by the insecticide was noted after 5, 10, 20, 30, 40, 45, 50 and 60 min. Mosquitoes of the susceptible reference strain of *An. gambiae* (Kisumu) were also subjected to the impregnated papers to ensure their quality. After 60 min of exposure, the mosquitoes were transferred to the observation tubes and provided a 10% honey solution and kept under observation for 24 h. After the tests, dead and live mosquitoes were kept separately in Eppendorf tubes containing silica gel and cotton and stored at − 20 °C for molecular characterization of resistance mechanisms and species.

### Molecular analysis

Female mosquitoes from susceptibility tube testing were analysed by PCR according to the protocols described by Scott et al. [22] and Favia et al. [23] for species and molecular forms identification respectively. The *kdr* L1014F mutation was also detected in the dead and live mosquitoes [24]. Only, a subset of mosquitoes (24–45 specimens randomly selected per district per year) was screened for molecular analyses.

### Statistical analysis

Data from this study was collected in two periods (Pre-intervention: in 2009 and, post-intervention: from 2012 to 2013). Mortality of *An. gambiae s.l.* following exposure to insecticides as well as the frequencies of the *kdr* L1014F mutation frequencies recorded during the two periods (2009 versus 2012–2013) were compared using a Chi square test. The same test was also used to compare *kdr* L1014F frequencies between dead and live mosquitoes exposed to deltamethrin, to investigate whether other mechanisms were implicated in resistance. A logistic regression followed by a maximum likelihood test was performed to assess whether the *kdr* L1014F mutation and, molecular species had an effect on the resistance status (dead or alive) of mosquitoes after susceptibility testing.

The association between Mmortality and *kdr* L1014F frequency was tested by calculating the risk ratio (RR), using the unconditional maximum likelihood estimation (Wald), and small sample adjustment (small). The RR Confidence intervals were determined using the normal approximation (Wald), and normal approximation with small sample adjustment (small), and bootstrap method (boot). Statistical analyses were performed with the R-2.15.2 software [25].

## Results

### Susceptibility of *An. gambiae* to insecticides pre- and post-intervention

**Fig. 2:**
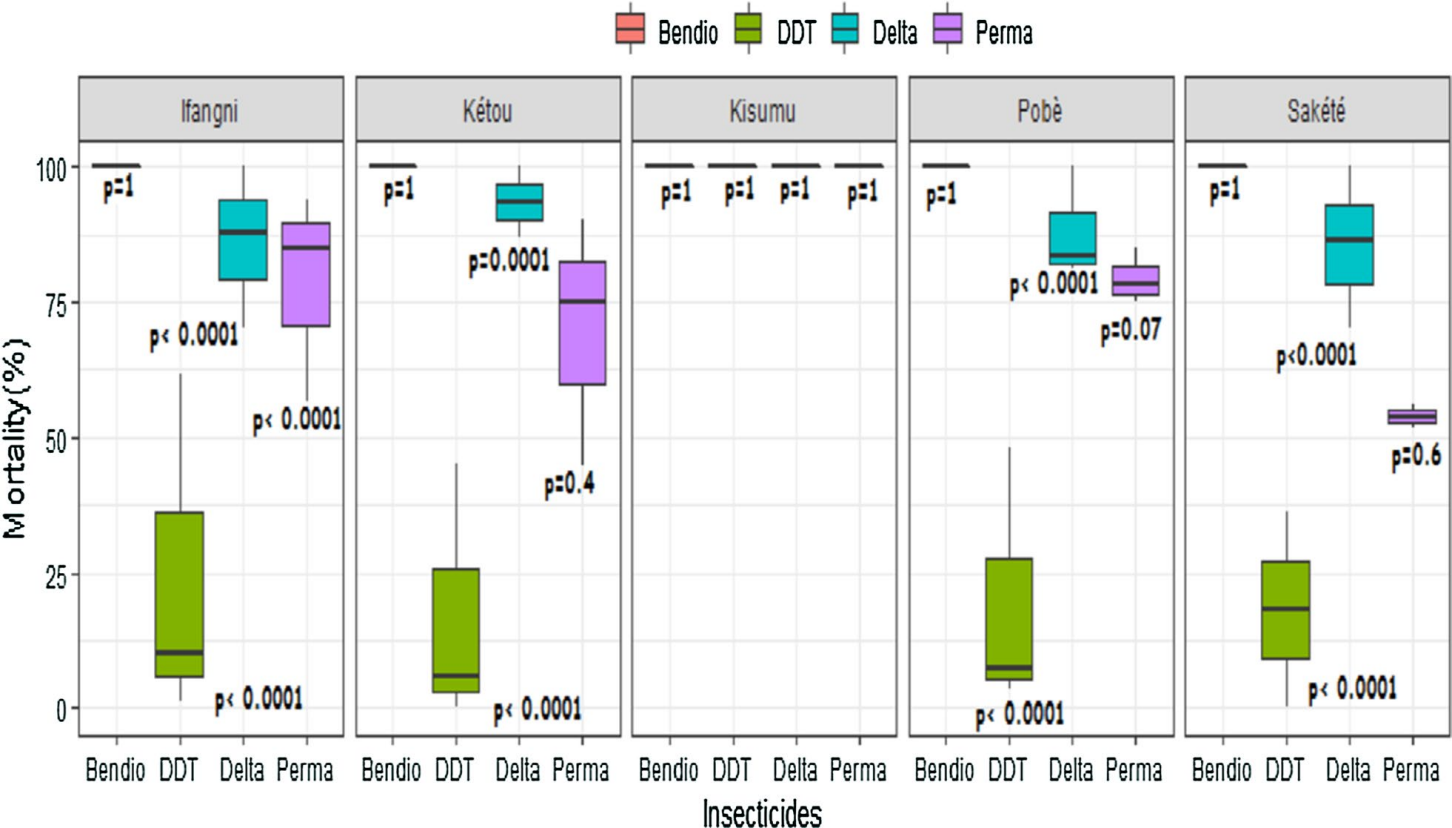
Variation in mortality rates of wild females *An. gambiae s.l.* and, of lab susceptible strain (*An. gambiae* Kisumu) to insecticides pre- and post-intervention. Bendio: Bendiocarb; Delta: Deltamethrin; Perm: Permethrin; DDT: Dichlorodiphenyltrichloroethane. The two lines outside the boxes are the whiskers

Throughout the study, the Kisumu reference strain was susceptible to all tested insecticides [deltamethrin (0.05%), permethrin (0.75%), bendiocarb (0.1%) and DDT (4%)] (Fig. 2). In all study districts, wild populations of *An. gambiae s.l*. were fully susceptible to bendiocarb with a 100% mortality rate recorded regardless of the period (pre- and post-intervention) (Fig. 2).

With deltamethrin, the mortality rate pre-intervention was 100% in all districts. However, this rate decreased to 78.9% (95% CI 73.3–84.5%) in Ifangni (p < 0.0001), 86.8% (95% CI 80.6–93%) in Kétou (p = 0.0001), 82.1% (95% CI 76.7–87.6%) in Pobè (p < 0.0001) and 78.1% (95% CI 72.2–84.1%) in Sakété (p < 0.0001) post-intervention (Fig. 2). Combining the data from the 4 districts, mortality rate of mosquitoes to deltamethrin decreased from 100% before intervention to 80.90% (95% CI 76.9–83.3%) after (p < 0.0001).

A similar trend was observed with permethrin in Ifangni where significant reduction in mortality rate was observed post-intervention [94% (95% CI 89.3–98.7%) vs 70.1% (95% CI 63.5–76.7%), p < 0.0001]. However, in the other three districts, although a slight decrease in susceptibility to permethrin was observed post-intervention, this effect was not significant [Kétou: 75% (95% CI 66.5–83.5%) vs 70.2% (95% CI 63.3–77.2%), p = 0.4], [Sakété: 56% (95% CI 46.3–65.7%) vs 51.7% (95% CI 39–64.3%), p = 0.6] and, [Pobè: 85% (95% CI 78–92%) vs 75.8% (95% CI 69.7–82%), p = 0.07] (Fig. 2). Combined data from the 4 districts show a significant decrease of mortality rate to permethrin post-intervention [77.5% (95% CI 73.4–81.6%) vs 70.1% (95% CI 67–73.2%), p = 0.01].

An increase in phenotypic resistance to DDT post-intervention was also observed in Ifangni (5.9%, 95% CI 2.3–9.4%), Sakété (0%), Pobè (4.4%, 95% CI 1.2–7.6%) and, Ketou (3.9%, 95% CI 0.5–7.3%) compared to the pre-intervention period where mortality rates were 62% (95% CI 52.5–71.5%, p < 0.0001), 36% (95% CI 26.6–45.4%, p < 0.0001), 48% (95% CI 38.2–57.8%, p < 0.0001) and, 45% (95% CI 35.2–54.8%, p < 0.0001), respectively (Fig. 2). Similarly, the combined data of the 4 districts show a decrease in the susceptibility of *An. gambiae s.l.* to DDT post-intervention [47.8% (95% CI 42.9–52.6%) vs 4.4% (95% CI 2.6–6.2%), p < 0.001].

### Frequency of the molecular species of the *An. gambiae* complex in the four study districts pre- and post-intervention

Table 1 shows the frequency of molecular species of the *An. gambiae s.l.* complex in the 4 study districts pre- and post-intervention. In total, 356 female *An. gambiae s.l.* collected over the study were analysed (Table 1). Pre-intervention (2009), only *An. coluzzii* had been detected in the four districts. The post-intervention (2012–2013) results revealed a proportion of 29.65% of *An. gambiae* and 70.35% of *An. coluzzii* after cumulating data of all 4 districts. The frequency of *An. gambiae* was of 7.14% in Ifangni, 20.83% in Sakété, 28% in Pobè and 65.52% in Ketou (Table 1).

**Table 1:** Frequency of molecular species by district according to the study period (pre and post intervention) in Benin

Districts	Periods	*Ag* s.l.	Molecular species			
			*Ac*	% *Ac*	*Ag*	% *Ag*
Ifangni	Pre-intervention	40	40	100	0	0
	Post-intervention	70	65	92.86	5	7.14
Sakété	Pre-intervention	30	30	100	0	0
	Post-intervention	48	38	79.17	10	20.83
Pobè	Pre-intervention	30	30	100	0	0
	Post-intervention	50	36	72	14	28
Kétou	Pre-intervention	30	30	100	0	0
	Post-intervention	58	20	34.48	38	65.52
Total	Pre-intervention	130	130	100	0	0
	Post-intervention	226	159	70.35	67	29.65

*Ag* s.l.: *Anopheles gambiae s.l*., *Ac: Anopheles coluzzii*, *Ag*: *Anopheles gambiae*

### Frequencies of the *kdr* L1014F mutation in the 4 study districts pre- and post-intervention

In the pre-intervention period, the *kdr* L1014F frequencies were relatively low in Ifangni [f(*kdr* L1014F) = 0.03] and Pobè [f (*kdr* L1014F) = 0.58] whereas they were very high in Sakété [f (*kdr* L1014F) = 0.90] and Ketou [f (*kdr* L1014F) = 0.92] (Table 2).

**Table 2:** Frequencies of the *Kdr* L1014F mutation in the 4 study districts pre- and post intervention in Benin

Districts	Periods	Total	Genotypes *Kdr L1014F*			F(*Kdr L1014F*)	x^2^-value	df	*p* value
			RR	RS	SS				
Ifangni	Pre-intervention	40	0	2	38	0.03^a^	186.36	1	< 0.0001
	Post-intervention	73	67	5	1	0.95^b^			
Sakété	Pre-intervention	30	24	6	0	0.90^a^	0.406	1	0.523
	Post-intervention	79	69	10	0	0.94^a^			
Pobè	Pre-intervention	30	11	13	6	0.58^a^	10.28	1	0.0013
	Post-intervention	80	55	19	6	0.81^b^			
Kétou	Pre-intervention	30	25	5	0	0.92^a^	0.0105	1	0.918
	Post-intervention	88	77	10	1	0.93^a^			
Total	Pre-intervention	130	60	26	44	0.56^a^	138.67	1	< 0.0001
	Post-intervention	320	268	44	8	0.91^b^			

^a,b^Values with different superscripts pre and post intervention within a same district are significantly different (p <  0.05)

Post-intervention, these frequencies were 0.94 in Sakété and 0.93 in Kétou and did not differ significantly compared to the pre-intervention period (p > 0.05) (Table 2). By contrast, a significant increase in the frequencies of the *kdr* L1014F mutation was observed post-intervention, in Ifangni [f (*kdr* L1014F) = 0.95, p < 0.0001] and Pobè [f (*kdr* L1014F) = 0.81, p = 0.0013] (Table 2). The evolution of this frequency was much more marked in Ifangni (0.03 pre-intervention vs. 0.95 post-intervention, p < 0.0001). Overall, by aggregating data from the 4 districts, the *kdr* L1014F frequency increased from 0.56 pre-intervention to 0.96 post-intervention (p < 0.0001) (Table 2).

### Frequencies of the *kdr* L1014F mutation in dead and live mosquitoes to deltamethrin in 2013

To evaluate the involvement of other mechanisms of pyrethroid resistance in *An. gambiae s.l.* from Plateau department, the *kdr* L1014F frequencies were compared between dead and live mosquitoes from susceptibility tests carried out with deltamethrin in 2013. In all districts, the results show that the *kdr* L1014F frequencies of dead mosquitoes were similar to those of live ones (p > 0.05) (Table 3). This result suggests that the *kdr* L1014F mutation is not the only mechanism involved in vector resistance to pyrethroids. However, the small numbers of mosquitoes tested in some cases did not allow for a representative estimate of the frequency of the *kdr* L1014F mutation.

**Table 3:** Frequencies of the *Kdr* L1014F mutation in dead and live mosquitoes to deltamethrin in Benin

Districts	Mosquito status	Total	Genotypes *Kdr* L1014F			F(*kdr*)	X^2^-value	df	p-value
			RR	RS	SS				
Ifangni	Dead	20	18	1	1	92.5^a^	1.38	1	0.239
	Live	20	20	0	0	100^a^			
Sakété	Dead	20	20	0	0	100^a^	n/a	1	1
	Live	12	12	0	0	100^a^			
Pobè	Dead	20	19	1	0	97.5^a^	< 0.0001	1	1
	Live	2	2	0	0	100^a^			
Kétou	Dead	20	20	0	0	100^a^	n/a	1	1
	Live	9	9	0	0	100^a^			
Total	Dead	80	77	2	1	97.5^a^	1.91	1	0.166
	Live	43	43	0	0	100^a^			

*n/a* no data

^a^Values with the same superscripts pre and post intervention within a same district are statistically similar (p  >  0.05)

### Assessment of the effect of *kdr* L1014F mutation and molecular species on the mortality of *An. gambiae* to deltamethrin in 2013

Table 4 shows the result of the logistic regression performed, to assess the effect of the *kdr* L1014F mutation and molecular species on the mosquito mortality to deltamethrin. Thus, no effect of *kdr* L1014F mutation (p = 0.273) and molecular species (p = 0.072) on the mosquito mortality is observed.

**Table 4:** Effect of *Kdr* L1014F mutation and molecular species on the mosquito mortality to deltamethrin in 2013 in Benin

Explanatory variables	OR (95% CI)	p (Wald’s test)	p (LR-test)
*Kdr* L1014F: RR	1	–	0.273
RS	8649436.23 (0, Inf)	0.992	
SS	8649436.23 (0, Inf)	0.995	
Molecular species: *An. gambiae* vs *An. coluzzii*	2.03 (0.93, 4.44)	0.076	0.072

### Evaluating the association between *kdr* L1014F and mortality rate in *An. coluzzii* and *An. gambiae*

In *An. coluzzii*, the *kdr* L1014F frequency was positively associated to the mortality rate (RR > 1) but, the association is not significant (p > 0.05) (Table 5). In *An. gambiae*, it was not possible to test the association as no RS or SS genotype was observed (Table 5).

**Table 5:** Association between the *Kdr* L1014F mutation and mortality to deltamethrin in 2013 in Benin

Molecular species	Genotypes (*Kdr* L1014F)	N (dead)	N tested	Mortality (%)	RR	CI-95% (RR)	p-value (Fisher test)
*An. coluzzii*	RR	37	65	56.9	1	–	–
	RS	2	2	100	1.8	1.4–2.2	0.5
	SS	1	1	100	1.8	1.4–2.2	1
*An. gambiae*	RR	40	54	74.1	1	–	–
	RS	0	0	ND	ND	ND	1
	SS	0	0	ND	ND	ND	1

*RR* risk ratio, *CI* confidence interval, *ND*: no data

## Discussion

The current study evaluated the evolution of insecticide resistance of malaria vectors in the Plateau department following mass distribution of Olyset nets in 2011 by the NMCP. Overall, *An. gambiae s.l*. was the main malaria vector in the Plateau department as previously showed by Padonou et al. [19] in Ouémé, a bordering department of Plateau. The molecular characterization revealed the simultaneous presence of *An. gambiae* and *An. coluzzii* post-intervention, whereas pre-intervention, only *An. coluzzii* was found. The detection of *An. coluzzii* only, over the pre-intervention period could presumably be due to the fact that, the data collection covered a shorter period as compared to the post-intervention period which spanned 2 years. This fully justified the low number of vector specimens sampled for PCR analysis pre-intervention. Thus, it is possible that *An. gambiae* were present at a very low frequency during the pre-intervention period and that a greater sample size of vectors may have demonstrated their presence. Moreover, the post-intervention data might have been generated with adult mosquitoes having emerged from larvae collected in highly diverse breeding sites as compared to the pre-intervention period. Hence, data collected during the post-intervention period may have provided a more representative capture of vector diversity and the species present in the study sites.

Post-intervention, the decrease in susceptibility to permethrin, deltamethrin and DDT combined with the significant increase in *kdr* L1014F frequency could be due to increased use of pyrethroid LLINs following the mass distribution by the NMCP in 2011. Similar observations have been made in Kenya and Niger, respectively by Stump et al. [26] and Czeher et al. [27]. Indeed LLINs might have killed susceptible mosquitoes within natural populations, thus selecting for resistant ones that will mate and produce more resistant offspring. Domestic use of aerosol insecticides [28] as well as the uncontrolled use of insecticides in agriculture [29] observed in Southern Benin, might have also been causal factors of increased pyrethroid resistance levels.

The decrease susceptibility to pyrethroid insecticides as well as the continued susceptibility to bendiocarb observed post-intervention in *An. gambiae s.l.* suggest that IRS with carbamate insecticides could effectively control *An. gambiae s.l.* in the Plateau Department. A combined intervention of pyrethroid LLINs and IRS with bendiocarb could be particularly effective in improving the impact of control whilst delaying the onset of resistance. However, the emergence of carbamate resistance in Atacora, a department in Northern Benin [30], emphasizes the importance of judicious insecticide application. Rotational use of IRS insecticides such as bendiocarb, pirimiphos-methyl and clothianidin, could prevent the establishment of resistance and preserve the effectiveness of the non-pyrethroid insecticide classes.

The logistic regression performed reveals that the *kdr* L1014F mutation as well as the molecular species were non-significantly correlated with the mortality rate to deltamethrin, which suggests that if they had an impact, it was in a very low way. The results are similar to those from Reimer et al. [31] in Cameroon. Thus, apart from the *kdr* L1014F mutation, a combination of other resistance mechanisms might explained the pyrethroid resistance observed in *An. gambiae s.l.* This is confirmed by the similarity of the *kdr* L1014F frequency in dead and live mosquitoes of the 4 surveyed districts. A non significant association between the *kdr* L1014F mutation and the mortality rate to deltamethrin was also observed in *An. coluzzii* while, the opposite result was obtained by Ibrahim et al. [32] with lambacyhalothrin.

The presence of mono-oxygenase mediated pyrethroid resistance has been demonstrated in *An. gambiae s.l.* collected in Missérété, a neighbouring site of the Plateau Department [33]. In addition, the presence of the N1575Y mutation was demonstrated in the natural populations of *An. gambiae s.l.* in Covè, another neighbouring district of the Plateau department [18]. It is, therefore, possible that the detoxification enzymes as well as the N1575Y mutation are also implicated in the resistance of the vectors to pyrethroids in the Plateau department.

## Conclusion

The data of the current study provide important information on vector resistance to insecticides in the Plateau Department, following mass deployment of LLINs. The decrease of the susceptibility of *An. gambiae s.l.* to pyrethroids and DDT, as well as the increase of the frequency of the *kdr* L1014F mutation constituted an alert to the NMCP which should at the time, consider development and implementation of an effective resistance management strategy. At the molecular level, it would have been of interest to perform insecticide resistance intensity tests, and Taqman PCR assays to evaluate the contribution of metabolic enzymes and N1575Y mutation to vector resistance to pyrethroids. At present, the effectiveness of a strategy for combatting malaria vectors in the area requires an update of their resistance status.

## Data Availability

All data generated or analysed during this study are included in this article and are available from the corresponding author.

## Abbreviations

NMCP: National Malaria Control Programme
LLIN: long-lasting insecticidal net
Kdr: knock down resistance
IRS: indoor residual spraying
CREC: Centre de Recherche Entomologique de Cotonou
WHO: World Health Organization
DDT: dichlorodiphenyltrichloroethane
PCR: polymerase chain reaction
